# Clinical Audit on the Effectiveness of Clinic-Based Intraocular Pressure Phasing for Patients With Glaucoma and Glaucoma Suspect

**DOI:** 10.7759/cureus.22726

**Published:** 2022-02-28

**Authors:** Selvaraja Nanthini, Ahmad Sukari Ain-Nasyrah, Raja Norliza Raja Omar, Azhany Yaakub, Ahmad Tajudin Liza-Sharmini

**Affiliations:** 1 Department of Ophthalmology and Visual Sciences, School of Medical Sciences, Health Campus, Universiti Sains Malaysia, Kubang Kerian, MYS; 2 Department of Ophthalmology, Hospital Universiti Sains Malaysia, Kubang Kerian, MYS; 3 Department of Ophthalmology, Hospital Melaka, Jalan Mufti Haji Khalil, Melaka, MYS

**Keywords:** intraocular pressure fluctuation, glaucoma, clinical audit, office hour phasing, diurnal intraocular pressure

## Abstract

Introduction: Glaucoma is a complex disease with intraocular pressure (IOP) playing an important role in its diagnosis and management. IOP has shown diurnal and nocturnal variations, which may affect the course of the disease.

Objective: The objective of this study was to determine the effectiveness of clinic-based office hour phasing in the diagnosis and management of glaucoma and glaucoma suspect (GS).

Methods: A retrospective clinical audit was conducted on patients who were subjected to office hour phasing in a glaucoma clinic, Hospital Universiti Sains Malaysia, Kelantan, Malaysia, between January 2015 and December 2019. The office hour phasing was conducted for various indications such as confirmation of diagnosis, screening, and effectiveness of treatment. IOP was recorded every two hours between 0800 and 1600 using an air puff tonometer by a trained nurse. Measurement of IOP was repeated with Goldmann applanation tonometer at sitting position by a trainee when the IOP ≥ 20 mmHg or discrepancy of IOP > 2 mmHg between two eyes. Other investigations including a Humphrey visual field and gonioscopy were conducted in between the IOP measurements. Diagnosis and clinical decisions on management were made at the end of the office hour clinic phasing.

Results: A total of 83 patients (163 eyes) were included in this clinical audit. Their mean age was 59.3 (16.5) years with 59% of male patients. Both eyes showed an almost similar pattern of mean IOP over five daytime readings in the clinic. A total of 35 eyes (21.5%) showed fluctuation ≥ 6 mmHg, and 128 eyes (78.5%) showed stable IOP during the clinic hour phasing. There was a significant difference in the mean IOP pattern between groups with stable and fluctuating IOP based on repetitive measure analysis of variance (RM ANOVA) (p = 0.008). The final diagnosis was made for 39 eyes (21 OD [right eye] and 18 OS [left eye]) out of 131 eyes (29.8%) with GS. Confirmation of diagnosis was achieved in all eyes (100%) with suspected ocular hypertension (OHT) and normal-tension glaucoma (NTG). Progression of glaucoma was confirmed in four eyes (2 OD and 2 OS) out of 17 eyes (23.5%) with suspected progression.

Conclusion: Clinic hour IOP phasing provides a practical approach in confirmation of diagnosis and adjustment in the management of patients with glaucoma and GS.

## Introduction

Glaucoma is defined as progressive optic neuropathy characterized by degeneration of retinal ganglion cells, which resulted in structural changes in the optic nerve head and subsequently visual field loss functionally [1]. Glaucoma causes irreversible blindness and is one of the main causes of blindness worldwide. Intraocular pressure (IOP) is identified as the only modifiable risk factor [2]. The level of IOP is part of the definition of certain types of glaucoma. IOP is also a significant risk factor for the progression of glaucoma [2,3]. However, IOP is found to be less sensitive and specific for the definitive diagnosis of glaucoma [3].

There are many factors affecting IOP, which include diurnal and nocturnal variations [4]. Diurnal variation of 2-6 mmHg is seen in normal individuals, and a higher variation is seen in patients with glaucoma [5]. Diurnal IOP fluctuation has been associated with the progression of glaucoma in some studies [6]. Recent studies suggested that the peaks, rhythm, and fluctuations of IOP have a part to play in glaucoma progression. A study with juvenile open-angle glaucoma (JOAG) patients showed that although patients were on optimal medical treatment and presented with apparently well-controlled IOP, they still experienced the temporary blurring of vision or progression of visual field defect [7]. These patients were subsequently found to have wider diurnal IOP variations.

Thus, a single snapshot IOP is inaccurate in formulating a diagnosis and assessing the effectiveness of the treatment. Sequential IOP assessment throughout a certain time period is known as phasing [8]. However, 24 hours IOP monitoring is not feasible as it requires hospital admission. Admission to the hospital is inconvenient to most patients as it disrupts their regular routine and incurs added costs. In addition, trained medical staff is required for IOP monitoring. Therefore, many ophthalmologists perceived that the information gained through phasing does not alter the management of their patients [8].

Office hour phasing may provide a practical method to study IOP variation and improve the accuracy in decision-making for glaucoma patients including glaucoma suspects (GSs) [9]. Phasing can be conducted on both treated and untreated patients. In untreated patients, phasing helps in diagnosis and screening for GS, normal-tension glaucoma (NTG), and ocular hypertension (OHT) [9]. While for patients with a confirmed diagnosis and on treatment, phasing helps to identify the impact of IOP fluctuation with the progression of glaucoma and assess the effectiveness of IOP-lowering medication [10]. The aim of this study is to determine the effectiveness of office hour phasing in the diagnosis and management of glaucoma in the sub-urban population.

## Materials and methods

A clinical audit was conducted on patients with glaucoma who were subjected to clinic-based IOP phasing between 1 January 2015 and 31 December 2019 in Hospital Universiti Sains Malaysia (HUSM), Kelantan, Malaysia. Patients who were diagnosed with GS, NTG, and OHT and those with clinical evidence of progression were subjected to clinic-based IOP phasing. Data of all patients who were identified for IOP phasing were recorded in a dedicated database in the clinic.

A GS is defined as a person who has one or more clinical features and/or risk factors that increase the possibility of developing glaucomatous optic neuropathy and visual deficiency in the future [9,11]. This includes those with elevated IOP, optic nerve head (ONH), or retinal nerve fiber layer (RNFL) appearance suggestive of glaucomatous damage, unexplained visual field (VF) defect consistent with glaucoma, and strong family history of severe glaucoma and other risk factors. NTG is an optic nerve neuropathy that presents with optic disk excavation and VF loss despite IOP < 21 mmHg [12]. OHT is defined as an eye with IOP > 21 mmHg, a normal optic nerve, and a normal visual field [11]. Clinic hour phasing was conducted for these patients to rule out any fluctuation of IOP, IOP trough and peak, that subsequently ascertains the final diagnosis. Any patient who was deemed to show signs of progression or at risk of progression clinically based on VF, optical coherent tomography (OCT), and fundus examination was also invited to be part of the clinic-based IOP phasing. Those who showed signs of progression but achieved target pressure are defined as adequate treatment and those who failed to achieve target pressure are regarded as inadequate treatment. The aim of the phasing of these patients is to determine if IOP fluctuation is the potential cause of progression that required a change in the management if deemed necessary.

A total of 165 patients were listed in the database. Fifty-three patients failed to turn up, and 29 patients failed to complete at least four IOP measurements. Clinic-based IOP phasing was conducted in the ophthalmology clinic, HUSM, between 0800 and 1630 hours. Patients were asked to come in early to the clinic and asked to wear light attire without a necktie or tight headscarf. They were asked to avoid any caffeinated drink for 24 hours and eat a light meal prior to the IOP phasing. They were also instructed to take their medications as per their regular prescription including topical pressure-lowering drugs.

IOP measurements were taken every two hours using air-puff tonometry (Reichert Inc., USA) at a sitting position by a trained nurse. All the five IOP measurements were taken by the same nurse. If the IOP is ≥ 20 mmHg, the second reading was taken using the Goldmann applanation tonometer (Haag-Streit International, Switzerland) at a sitting position by an ophthalmology trainee. For each patient, the tonometer was disinfected routinely. The tonometer was calibrated weekly as per the procedure. At the final IOP reading, gonioscopy was performed using a Goldmann 2-mirror contact lens (OCULAR Instruments Inc., USA) to ascertain if the anterior chamber angle was open. Patients then had a slit lamp (Topcon, Japan) examination of the optic disk and peripapillary area.

During the clinic-based IOP phasing, other relevant investigations were conducted, which include VF assessment using Humphrey Field Analyzer II (Carl Zeiss, USA), OCT on optic nerve head (ONH), central corneal thickness (CCT), and refraction in two-hour intervals. This allowed clinical decisions to be made at the end of office hour phasing by a glaucoma specialist (LS and AY). The clinical decision was divided into confirmation of diagnosis, progression, and change in treatment. IOP peak and trough of both eyes were identified for clinical and statistical analyses.

The clinical decision was derived based on individual IOP measurement and/or the presence of ≥ 6 mmHg of fluctuation between peak and trough. For example, any GS patients that showed the fluctuation of ≥6 mmHg or at least two readings that were above 21 mmHg were analyzed and diagnosed accordingly as primary open-angle glaucoma (POAG), OHT, or remain as GS. If any patients were initially diagnosed as NTG but showed evidence of raised IOP (>21 mmHg) or fluctuation of IOP of ≥6 mmHg, their diagnoses were revised. For example, if there were two IOP measurements of 24 mmHg and 23 mmHg in a patient with NTG but the fluctuation of IOP < 6 mmHg, the diagnosis for this patient was revised. Similarly, if there was a patient who demonstrated IOP fluctuation ≥ 6 mmHg but his/her IOP measurements were between 11 mmHg and 18 mmHg, the diagnosis will not change.

Change of management was advocated for patients with structural or functional evidence of glaucoma progression and also for those patients who showed evidence of IOP fluctuation of ≥6 mmHg based on clinic hour IOP phasing. The diagnosis was not made solely based on the clinic hour phasing but with supporting structural and functionally glaucomatous optic neuropathy and other risk factors. For analysis purposes, the IOP diurnal pattern was also studied, and they were divided into two groups: stable IOP and fluctuation of IOP.

All data were entered into the Statistical Program for Social Science (SPSS) version 22.0 software (IBM Corp, Armonk, NY). They were checked and cleaned to ensure accurate documentation and to eliminate any missing or erroneous values. Comparison between the right eye (OD) and left eye (OS) was made using paired t-test. Repetitive measure analysis of variance (RM ANOVA) was used to compare between the eyes with stable IOP and fluctuation of IOP. P-value < 0.05 was deemed statistically significant in this study.

## Results

**Figure 1:**
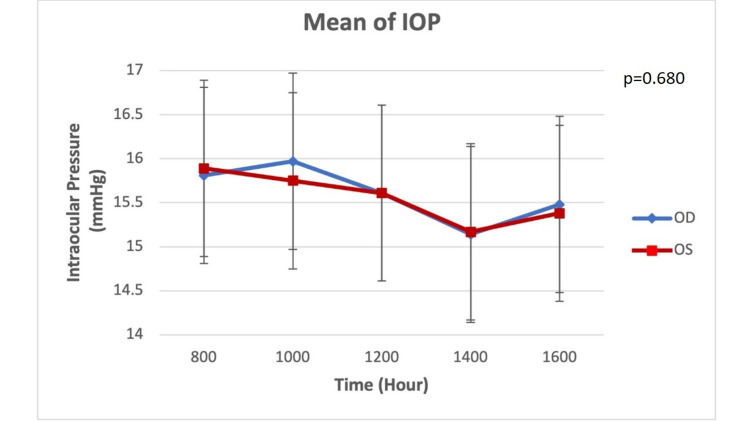
Mean IOP for OD and OS during office hour phasing based on paired T-test IOP: Intraocular pressure; OD: Right eye; OS: Left eye.

A total of 83 patients (163 eyes) were included in this clinical audit. Clinic-based phasing was conducted on bilateral eyes in 80 patients and unilateral eyes in three patients. The unilateral cases were precious eyes (OS), while the other eyes were blind due to glaucoma. Their mean age was 59.3 (16.5) years with 59% men (Table 1). Both eyes showed an almost similar pattern of mean IOP over five daytime readings in the clinic (Figure 1). In general, the IOP taken between 0800 and 1200 was higher than 1400 and 1600. Both eyes showed the lowest mean IOP at 1400 hours and highest at 0800 hours. The frequency of eyes according to the time of peak IOP also showed a similar pattern (Tables 2, 3). A higher frequency of trough IOP was seen between 1400 and 1600 hours (Table 2).

**Table 1:** Demographic data

Characteristics	n (%)
Age (mean, SD)	59.2 (16.5)
Sex (n, %)	
Male	49 (59.0)
Female	34 (41.0)
Race (n)	
Malay	67 (80.7)
Chinese	14 (16.9)
Indian	1 (1.2)
Others	1 (1.2)
Systemic diseases (n)	
Diabetes mellitus	50 (60.2)
Hypertension	46 (55.4)
Ischemic heart disease	7 (8.4)
Hyperlipidemia	19 (22.9)
Renal diseases	4 (4.8)
Others	9 (10.8)
Distance from the hospital	
<5 km	17 (20.5)
5-15 km	34 (41.0)
>15 km	32 (38.6)

**Table 2:** Distribution of mean, peak, and trough IOP according to the clinic hour phasing

	Mean IOP (SD)		Mean Peak IOP (SD)		Mean Trough IOP (SD)	
Time	OD (n = 80)	OS (n = 83)	OD (n = 80)	OS (n = 83)	OD (n = 80)	OS (n = 83)
800	15.8 (4.2)	15.9 (4.6)	19.2 (5.1)	20.3 (5.3)	13.7 (3.4)	13.1 (2.1)
1000	15.6 (4.2)	15.8 (4.2)	18.2 (4.8)	16.8 (3.7)	14.8 (3.2)	14.3 (4.6)
1200	15.6 (4.2)	15.6 (3.6)	19.8 (4.0)	17.2 (4.1)	13.9 (3.7)	18.2 (4.4)
1400	15.1 (3.8)	15.2 (4.1)	18.6 (4.3)	16.2 (3.9)	13.7 (2.9)	13.8 (2.7)
1600	15.5 (3.8)	15.4 (4.1)	16.1 (3.3)	19.8 (5.5)	13.6 (2.0)	13.8 (3.2)
Mean	15.6 (4.0)	15.6 (4.1)	18.4 (4.3)	18.1 (4.5)	13.9 (3.0)	14.7 (3.4)

**Table 3:** Distribution between the frequency of peak and through IOP according to the clinic hour phasing

	Frequency of OD (n = 80)		Frequency of OS (n = 83)	
Time	Peak IOP (n, %)	Trough IOP (n, %)	Peak IOP (n, %)	Trough IOP (n, %)
800	24 (28.9)	14 (17.5)	23 (27.7)	11 (13.3)
1000	20 (25.0)	10 (12.5)	21 (25.3)	15 (18.1)
1200	13 (16.3)	20 (25.0)	17 (20.5)	17 (20.5)
1400	7 (8.8)	20 (25.0)	10 (12.0)	15 (18.0)
1600	16 (20.0)	16 (20.0)	12 (14.5)	25 (30.0)

**Figure 2:**
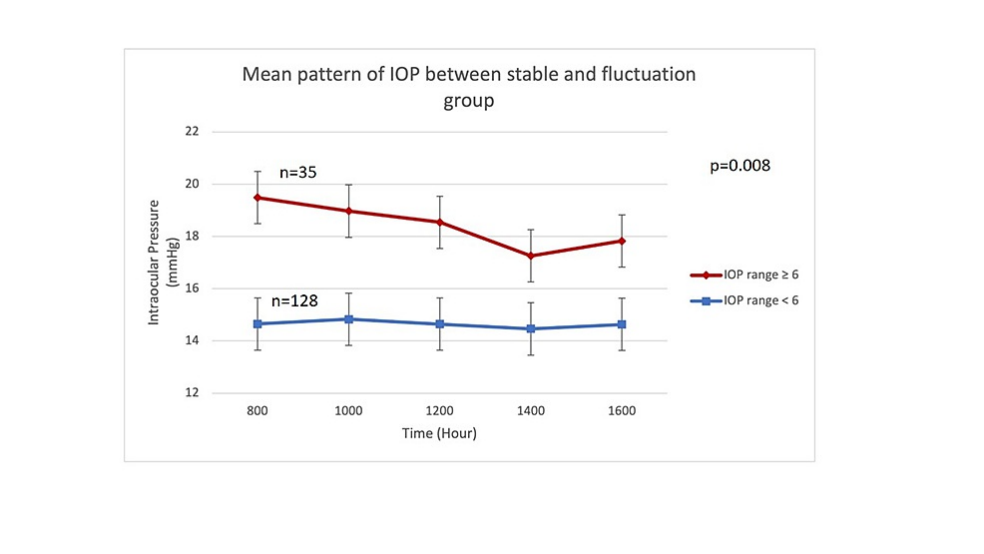
Mean pattern of IOP between stable and fluctuation groups based on RM ANOVA IOP: Intraocular pressure; RM ANOVA: Repetitive measure analysis of variance.

In general, the group with fluctuation showed higher baseline IOP (0800), and the range of fluctuation was wider. A total of 35 eyes (21.5%) showed fluctuation ≥6 mmHg, and 128 eyes (78.5%) reflected stable IOP during the clinic hour phasing. There was a significant difference in the mean IOP pattern between groups with stable and fluctuating IOP based on RM ANOVA (p = 0.008) (Figure 2).

Table 4 shows the indication and final clinical decision of eyes that were subjected to clinic IOP phasing. The final diagnosis was made for 39 eyes (21 OD and 18 OS) out of 131 eyes (29.8%) with GS. Confirmation of diagnosis was achieved in all eyes (100%) with suspected OHT and NTG. Progression of glaucoma was confirmed in four eyes (2 OD and 2 OS) out of 17 eyes (23.5%) with suspected progression.

**Table 4:** Comparison of diagnosis pre- and post-clinic hour phasing for the right and left eyes OD: Right eye; OS: Left eye; VCDR: Vertical cup to disc ratio; IOP: Intraocular pressure; FH: Family history of glaucoma; GS: Glaucoma suspect; OHT: Ocular hypertension; NTG: Normal-tension glaucoma; POAG: Primary open-angle glaucoma; PACG: Primary angle-closure glaucoma.

OD (n = 80)				OS (n = 83)		
Indication		Pre-phasing	Post-phasing	Indication	Pre-phasing	Post-phasing
							Glaucoma suspect		Diagnosis based on	Final diagnosis	Glaucoma Suspect	Diagnosis based on	Final diagnosis
(n = 65)		VCDR: 24 (36.9)	GS: 44 (67.7)		VCDR: 26 (39.4)	GS: 48 (72.7)
		IOP: 38 (58.5)	OHT: 5 (7.7)	(n = 66)	IOP: 37 (56.1)	OHT: 2 (3.0)
		FH: 3 (4.6)	NTG: 7 (10.8)		Fam hx: 3 (4.5)	NTG: 8 (12.2)
			POAG: 8 (12.3)			POAG: 7 (10.6)
			PACG: 1 (1.5)			PACG: 1 (1.5)
Confirmation of diagnosis		OHT: 4 (66.6)	OHT: 0 (0)	Confirmation of diagnosis	OHT: 7 (77.8)	OHT: 2 (22.2)
(n = 6)		NTG: 2 (33.4)	NTG: 3 (50.0)	(n = 9)	NTG: 2 (22.2)	NTG: 2 (22.2)
			POAG: 3 (50.0)			POAG: 5 (55.6)
Progression		Adequate: 5 (55.6)	Progress: 2 (22.2)	Progression	Adequate: 4 (50.0)	Progress: 2 (25.0)
(Treatment)		Inadequate: 4 (44.4)	Non-progress: 7(77.8)	(Treatment)	Inadequate: 4 (50.0)	Non-progress: 6 (75.0)
(n = 9)				(n = 8)		

## Discussion

The effectiveness of a single IOP measurement versus 24 hours IOP monitoring has been debated on numerous occasions. On the basis of the influence of diurnal and nocturnal variations, many ophthalmologists believe in conducting IOP measurements for 24 hours [13]. However, this is less practical and inconvenient to the patients [9]. Thus, there are recommendations to conduct multiple measurements during office hours (diurnal) in a clinic setting. There is supportive evidence to suggest that daytime serial IOP is similar to the night-time measurement [13,14]. Moodie et al. found that 24 hours measurement did not confer any extra advantage as compared to daytime IOP measurement in patients with evidence of glaucoma progression. Daytime measurement was similar to night-time measurement [8]. However, Hughes et al. found that there was no significant difference in the mean IOP at daytime and 24 hours measurement, but the ability to identify peak IOP was significantly higher with 24 hours measurement [15].

In this study, we found that daytime phasing during clinic hours is well accepted by the patients. Even those who were staying >15 km away (38.6%) from the hospital were willing to participate. Our protocol of the clinic hour phasing allows the relevant investigation to be conducted in between the IOP measurements. It is practical and did not cause any inconvenience to the participants.

In general, our patients reflected a distinctive diurnal variation with higher IOP measurements in the morning (0800-1200) and lower IOP in the afternoon (1400-1600). There was a dip in the reading at 1400. A similar diurnal pattern was reported on normal subjects, POAG, and NTG patients [16,17]. In general, the diurnal pattern of IOP demonstrated in this clinical audit is almost similar to the studies conducted in Europe [18,19]. There was no significant difference in the IOP pattern between the two eyes. Thus, suggesting in the cases of bilateral eyes involvement, the pattern of IOP is potentially the same between both eyes. However, this clinic-based phasing may not be able to detect night-time fluctuation and the highest mean peak. This is perhaps important for glaucoma with wide fluctuation such as pseudoexfoliation glaucoma (PXG) and PACG. In addition, lateral decubitus position affects the IOP measurement especially at night [18]. Thus, our knowledge of night-time IOP is limited. Conducting 24-hour IOP in the sleep lab or ward may not accurately represent the actual situation. IOP is affected by position, and lateral decubitus position causes higher IOP measurement [18].

Diurnal and nocturnal IOP is affected by the type of glaucoma, and an individual variation has also been documented [19,20]. It was also reported that this variation is based on the observation that was carried out over a period of 48 hours rather than 24 hours [21]. A longer duration of serial IOP measurement is ideal but may not be practical in developing countries such as Malaysia. Perhaps, a longer duration of IOP phasing should be reserved for specific cases when the clinic hour phasing failed to detect the variation. Based on our findings, there were patients who did not show any IOP variation (stable) and some who showed fluctuation. In general, those who developed fluctuation demonstrated a sudden dip at 1400 and highest at 0800. Thus, defining the diagnosis based on the difference in IOP variation is not robust enough without considering other factors. The strength of this clinical audit, the diagnosis, and changes of treatment were based on the actual clinical setting.

However, clinic IOP phasing was only able to aid less than a third of our clinical decisions. Cases of GS are challenging in many clinical practices especially when there is no proper reference guidance [22,23]. Based on Clinical Practice Guideline 2017, a thorough evaluation including diurnal IOP and identification of risk factors are recommended [24]. Other risk factors were also considered before any clinical decision is made in the present study. In this clinical audit, IOP phasing had helped to confirm the diagnosis of glaucoma in 29.8% of patients who are referred as glaucoma suspects. The majority of them were still labeled as GS, while detection of progression of glaucoma was only achieved in 23.5% of cases. However, based on this audit, confirmation of diagnosis for OHT and NTG was achieved in all cases. Serial IOP measurement is essential for cases with OHT and NTG [11,25,26]. The sensitivity and specificity of this method are not the main aims of this audit.

Office hour phasing is critical in early detection and helped with appropriate management of patients with glaucoma or suspected to have glaucoma, despite it being time-consuming for both patients as well as clinicians. Therefore, office hour phasing may not be ideal but better than relying on only a single IOP to confirm the diagnosis, especially in the cases of NTG and OHT. A single snapshot IOP measurement may lead to misinterpretation and unnecessary or late initiation of treatment. The availability of digital sensors for continuous IOP measurement will be the way forward to improve detection and prevention of delay in the management of patients with glaucoma [6,27,28]. Daily activities of patients with glaucoma may cause fluctuation of IOP and have a detrimental effect, especially those in the advanced stage of the disease [28,29]. Emotional distress was also identified to cause elevation of IOP using contact lens sensor telemetry. In this study, the assumption was made without considering the effect of daily activities and emotional distress on IOP.

## Conclusions

In conclusion, clinic hour IOP phasing is practical and convenient for patients with glaucoma and GS. Clinical hour IOP phasing provides a more reliable guide in the confirmation of diagnosis and the need to change the management. Serial IOP phasing should be individualized according to the patient’s needs.
